# Subtle elbow instability associated with lateral epicondylitis

**DOI:** 10.1186/s12891-018-2069-8

**Published:** 2018-05-07

**Authors:** Sang Ho Kwak, Seung-Jun Lee, Hee Seok Jeong, Min Uk Do, Kuen Tak Suh

**Affiliations:** 1 Department of Orthopaedic Surgery, Pusan National University Yangsan Hospital, Pusan National University School of Medicine, 20, Geumo-ro, Mulgeum-eup, Yangsan-si, Gyeongsangnam-do Republic of Korea; 2 Department of Radiology, Pusan National University Yangsan Hospital, Pusan National University School of Medicine, 20, Geumo-ro, Mulgeum-eup, Yangsan-si, Gyeongsangnam-do Republic of Korea

**Keywords:** Lateral, Epicondylitis, Tendinosis, Ligament, Instability, Elbow

## Abstract

**Background:**

In lateral epicondylitis, even in the absence of apparent instability, subtle instability can be found under anesthesia. We wanted to ascertain the following: (1) how many elbows surgically treated with lateral epicondylitis showed subtle instability during examination under anesthesia (EUA), (2) how effective magnetic resonance imaging (MRI) was in predicting subtle instability, and (3) if any difference existed in preoperative clinical data between elbows with and without subtle instability during EUA.

**Methods:**

One hundred and twenty-two elbows (117 patients) diagnosed with intractable lateral epicondylitis underwent surgical treatment. No elbow showed apparent instability with conventional physical examination. Under general anesthesia, the elbows were examined for subtle instability via fluoroscopy and divided into unstable and stable groups. Potential prognostic factors and functional scores were assessed retrospectively. The MRIs were reviewed again by two radiologists.

**Results:**

Seventeen elbows (unstable group, 13.9%) had subtle instability in EUA, while 105 elbows (stable group, 86.1%) did not. Lateral collateral ligament (LCL) complex injury was noted in the MRIs of 28 elbows. Fifteen elbows showed subtle instability among 28 elbows with abnormal MRI (positive predictive value, 53.6%), while 81 elbows did not show subtle instability among 82 elbows with normal MRI (negative predictive value, 98.7%). The preoperative visual analog scale score was higher in the unstable group than in the stable group (*p* < 0.001), and a history of multiple corticosteroid injections (≥3) was related to subtle instability in EUA (*p* = 0.042). Other factors showed no significant differences between both groups.

**Conclusions:**

Subtle instability resulting from LCL complex injury was noted in elbows with lateral epicondylitis. This could be visualized with fluoroscopic EUA, and preoperative MRI could be used to exclude subtle instability. Surgeons should consider checking for subtle instability, especially when patients have a history of multiple corticosteroid injections (≥3) or severe pain and MRI indicates instability.

## Background

Lateral epicondylitis is usually diagnosed based on clinical history and physical examination. Excluding conditions that can mimic lateral epicondylitis is very important because symptoms cannot be fully relieved if such lesions are neglected. For a differential diagnosis, multiple modalities including simple radiography, ultrasonography, magnetic resonance imaging (MRI), and electrodiagnosis can be used.

Associated ligament injuries in lateral epicondylitis were reported using MRI or via conventional physical examination. MRI can be used for differentiating other pathologic conditions such as plica, elbow arthritis, and osteochondral defect. In addition, concomitant ligament injuries involving the medial collateral ligament (MCL), lateral collateral ligament (LCL), and lateral ulnar collateral ligament (LUCL) have also been observed in MRI-diagnosed lateral epicondylitis [1, 2]. Associated ligament injuries can induce elbow instability eventually, and posterolateral rotatory instabilities after trauma, corticosteroid injection, and iatrogenic injury during debridement for lateral epicondylitis have been reported [3, 4]. In these reports, most of the patients showed apparent symptoms including instability via conventional physical examination, feeling of “pop,” or prominent swelling of the elbow joint [3, 4]. However, Kalainov revealed that one patient showed unspecific physical examination and the posterolateral instability was detected only during general anesthesia [5]. Morrey et al. described lax LCL and LUCL found in fluoroscopy under local anesthesia or arthroscopic examination as subtle instability, which the authors reported as causes of refractory lateral epicondylitis [4]. Thus, even though conventional physical examination may not reveal associated ligament injuries, subtle instability can be found under specific examination with anesthesia. However, reports of subtle instability found in primary surgery for lateral epicondylitis are very rare, and except for one case [5], only one study reported about subtle instability under anesthesia and suggested a treatment algorithm [6].

In this study, we examined elbow instability in fluoroscopy during examination under anesthesia (EUA) and aimed to report the elbows that needed primary surgical interventions for lateral epicondylitis: (1) how many elbows had subtle instability, (2) how closely the EUA findings matched the MRI and operative findings in elbows with subtle instability, and (3) whether any differences existed regarding preoperative clinical data between elbows with and without subtle instability.

## Methods

This was a retrospective case series study. After approval by our institutional board review (IRB number 05–2017-028), 173 consecutive elbows (168 patients) with lateral epicondylitis treated surgically between March 2011 to December 2016 were enrolled in this study. Definitive criteria for lateral epicondylitis included 1) pain at the elbow during the preceding 30 days and 2) pain at the lateral humeral epicondyle region and pain provoked by resisted extension of the wrist with the elbow extended [7]. All patients were treated with a combination of four conservative methods including NSAID administration, counterforce bracing, isometric exercise, and extensor muscle stretching. Injections including corticosteroid, autologous blood, or botulinum toxin were not given in our protocol. The surgical indication was intractable pain (visual analog scale [VAS] ≥ 4) after conservative treatment for at least 6 months. Simple radiographs were taken at initial diagnosis, and after deciding to treat with surgery, MRIs were performed. Elbows with previous trauma, including elbow dislocation and fracture (*n* = 17); deformities, including cubitus varus and valgus (*n* = 11); synovial plica and osteochondral defect on MRI (*n* = 5); and previous surgery for lateral epicondylitis (*n* = 7) were excluded. The mediolateral stress, posterolateral rotatory drawer, push-up, and tabletop tests were conducted again by two orthopedic specialists (KSH, LSJ) before surgery. If at least one examiner found any instability in these examinations, the patients (*n* = 8) were considered to have instability during conventional physical examination and excluded. None of the patients showed generalized ligament laxity (Beighton score < 4) [8]. Patients detected with synovial plicae intraoperatively (*n* = 3) were also excluded. Finally, 122 elbows (117 patients) were included in this study.

### Identifying instability during EUA

**Fig. 1:**
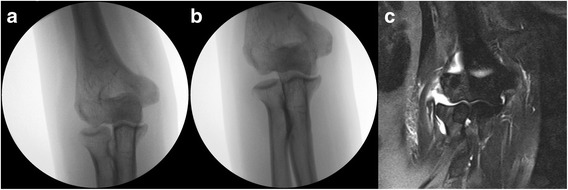
**a** Intraoperative fluoroscopy image of a 53-year-old woman (patient #1). **b** Widening of the radiocapitellar joint was identified 2.3 mm in the varus stress view. **c** T2-weighted coronal magnetic resonance image showed rupture of the lateral collateral ligament and tear of the extensor tendon origin

**Fig. 2:**
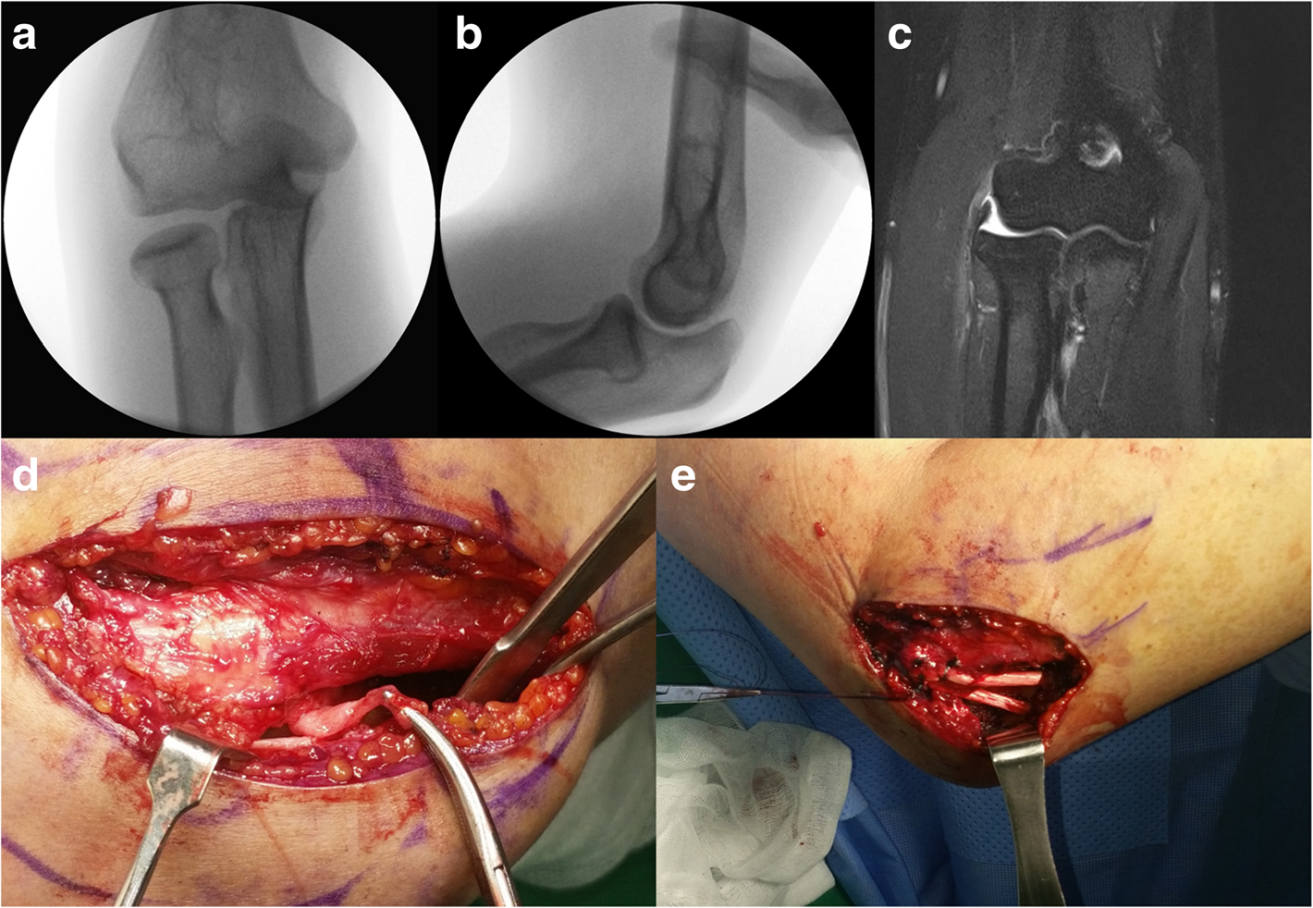
**a** The 2.1 mm widening of the radiocapitellar joint was identified in the varus stress view of a 38-year-old man (patient #5). **b** The radial head was shifted posteriorly in the posterolateral pivot shift test. **c** T2-weighted coronal magnetic resonance image showed rupture of the lateral collateral ligament. **d** Intraoperatively, the definite lateral and lateral ulnar collateral ligaments were not found, except for thin fibrous tissue. **e** The lateral ulnar collateral ligament was reconstructed with a tibialis anterior allograft

The patients were placed in the supine position under general anesthesia. For valgus instability, the arm was positioned with the elbow in 30° flexion, humerus in full internal rotation, and forearm in pronation. Then, the manual valgus stress test was performed, and an anteroposterior (AP) image of the elbow was acquired using a fluoroscopic image intensifier [9]. A widening of more than 1 mm at the ulnohumeral joint was considered as subtle valgus instability [9]. For varus instability, the arm was positioned with the elbow in 15° flexion, humerus in full external rotation, and forearm in supination. Then, manual varus stress test was performed, and an AP fluoroscopic image was acquired. Since a previous study reported that the radiocapitellar joint is 0.47 mm more redundant compared with the ulnohumeral joint [10], a widening of over 1.5 mm at the radiocapitellar joint was considered as subtle varus instability (Fig. 1). In addition, a posterolateral pivot shift test was performed with the elbow in 90° flexion and forearm in supination [11]. If the longitudinal axis of the radius does not pass through the center of the capitellum, it was considered as a posterolateral instability [9]. Thereafter, a lateral image of the elbow was acquired to identify subtle posterolateral instability (Fig. 2a-c) [5]. If subtle instability was found, the contralateral asymptomatic elbow was examined to exclude underlying normal laxity of elbow joint.

### Surgical exploration of ligament status

During surgery, intraarticular pathology was checked via arthrotomy, and 3 patients with synovial plicae were excluded. For patients without subtle instability, we performed open release of lateral epicondylitis. The LCL complex was not explored to minimize damage to the extensor tendon. For patients with subtle instability, additional exploration for unstable ligament was performed. For varus or posterolateral instability, the proximal LCL complex was explored by elevating the common extensor origin, and the distal LCL complex was explored by elevating the anconeus and extensor carpi ulnaris. For valgus instability, the MCL was explored via an additional medial longitudinal incision.

### Preoperative clinical data and MRI evaluation

Demographic data that were considered as potential prognostic factors such as patient age, gender, duration of education, and involvement of the dominant hand were assessed retrospectively. Other potential prognostic factors such as duration of symptoms, smoking, body mass index (BMI), and history of multiple corticosteroid injections (≥3) were also assessed [7, 12–15]. Preoperatively, VAS score, Mayo Elbow Performance Score (MEPS), and Quick Disabilities of the Arm, Shoulder, and Hand (Quick DASH) score were assessed by an experienced orthopedic surgeon who did not attend the surgery. To identify ligament lesions in MRI, two radiologic specialists (TYM, HSJ), who had no knowledge of the intraoperative findings, reviewed the MRIs again.

### Statistical analysis

Data were presented as mean ± standard deviation (SD). Categorical variables were analyzed using Fisher’s exact test, and continuous variables were analyzed using the Mann-Whitney test. Statistical significance was defined as a *P*-value< 0.05. Data were analyzed with SPSS for Windows version 18.0 (SPSS, Chicago, Illinois, USA).

## Results

Of the 122 elbows (117 patients), 17 elbows (16 patients) with subtle instability were assigned to the unstable group during EUA. One hundred and five elbows (101 patients) without instability were assigned to the stable group. All 17 elbows in the unstable group showed varus instability (radiocapitellar joint widening; range, 2.5~ 4.1 mm), and two showed additional posterolateral instability. No elbow in the unstable group showed valgus instability under EUA (ulnohumeral joint widening; range, 0.13~ 0.66 mm). Except for 1 patient in the unstable group (patient 2), there was no patient who showed subtle instability on the contralateral asymptomatic elbow. In 105 elbows without subtle instability, the radiocapitellar and ulnohumeral joint widening was measured, ranging from 0.45 to 1.32 mm and 0.15 to 0.75 mm, respectively.

**Fig. 3:**
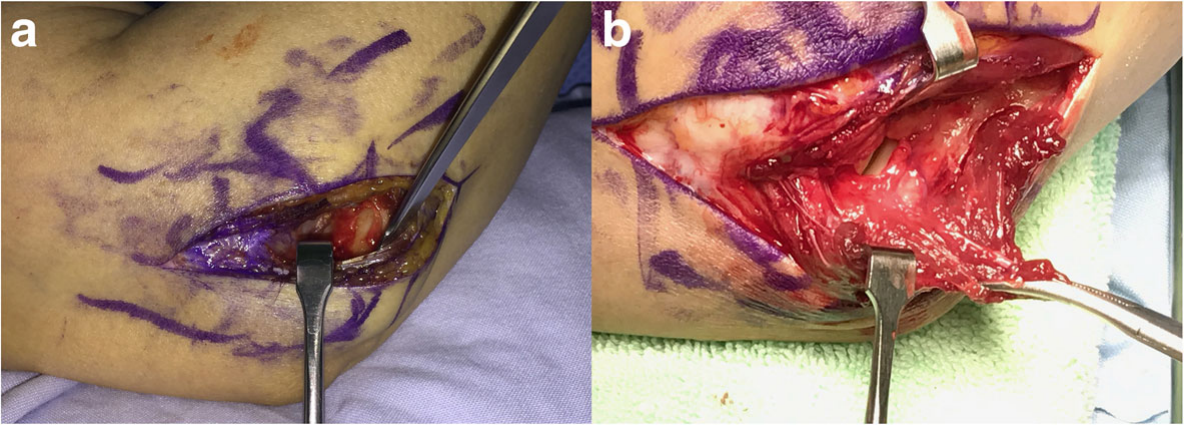
**a** Example of a deficient LCL complex: no visible functioning LCL complex was found. **b** Example of partial defect of the LCL complex: there was a defect in the LCL complex, but a functional LCL complex partially remained

One elbow in the unstable group and 11 elbows in the stable group did not undergo MRI for economic reason. Among 16 elbows which underwent MRI in the unstable group, 4 LCL + LUCL injuries, 6 LUCL injuries, and 5 LCL injuries were found. In 1 patient (patient 8), there was no notable ligament tear except for signal change on MRI taken preoperatively. In the stable group, 94 elbows underwent MRI, and 8 LCL and 5 LUCL injuries were noted on MRI. A combined LCL + LUCL injury was not identified. In both groups, no MCL injury was noted with MRI. Of all 28 elbows with LCL complex injuries on MRI, 15 showed subtle instability with EUA. Intraoperatively, in the unstable group, LCL complex abnormalities were confirmed at surgery in all cases; no functioning LCL and LUCL structure except the annular ligament was considered as a deficient LCL complex (*n* = 8) and remained a functioning structure despite that the defect or attenuation was considered as a partial loss of the LCL complex (*n* = 9) (Fig. 3). Capsules were not torn but attenuated in all patients (Table 1).

**Table 1:** Patient demographics, findings in MRI and EUA, and complication in unstable group

Patient	Gender	Age (decade)	Handedness	Duration of symptom	Multiple Injection history	Instability under EUA	MRI	Intraoperative findings of LCL complex
1	F	6th	Rt(D)	7	+	Var	LCL, LUCL	Deficient
2	F	6th	Rt(D)	24	+	Var	LUCL	Deficient
			Lt(ND)	24	+	Var	LUCL	Partial defect
3	M	6th	Rt(D)	36	+	Var,	LCL	Deficient
4	M	5th	Rt(D)	6	–	Var, Posterolat	LCL, LUCL	Partial defect
5	M	4th	Rt(D)	24	+	Var, Posterolat	LUCL	Deficient
6	F	6th	Rt(D)	12	+	Var	LUCL	Partial defect
7	F	7th	Lt(ND)	12	+	Var	LUCL	Deficient
8	M	5th	Rt(D)	10	+	Var	intact	Partial defect
9	F	5th	Lt(ND)	14	+	Var	LCL	Partial defect
10	F	5th	Lt(ND)	12	+	Var	LUCL	Deficient
11	F	6th	Rt(D)	60	+	Var	LCL	Deficient
12	M	5th	Rt(D)	10	+	Var	LCL	Partial defect
13	F	5th	Rt(D)	12	+	Var	Not taken	Partial defect
14	M	6th	Lt(ND)	14	+	Var	LCL	Partial defect
15	M	5th	Rt(D)	12	+	Var	LCL, LUCL	Partial defect
16	M	4th	Rt(D)	12	+	Var, Posterolat	LCL, LUCL	Deficient

*EUA* Examination under anesthesia, *MRI* Magnetic resonance image, *LCL* Lateral collateral ligament, *LUCL* Lateral ulnar collateral ligament

Duration of symptoms, gender, age, dominant hand involvement, occupation, educational periods, smoking, BMI, preoperative MEPS, and preoperative Quick DASH score were not different between both groups. The multiple corticosteroid injection rate (≥3) and preoperative VAS score were higher in the unstable group than in the stable group (Table 2).

**Table 2:** Comparison of preoperative clinical data. Continuous variables were presented as mean (standard deviation)

	Unstable Group	Stable Group	*P*-value
Gender(M/F)	8/9	45/60	0.473
Age (year)	48.9 (6.61)	50.5 (7.47)	0.392
Dominant/Nondominant	12/5	65/40	0.344
Manual worker(+/−)	9/8	46/59	0.329
Duration (month)	17.7 (13.3)	14.5 (10.3)	0.321
Eductaion (year)	12.6 (2.18)	11.9 (2.37)	0.275
Multiple corticosteroid injection(> 3)(+/−)	16/1	76/29	0.042^*^
Smoking(+/−)	7/10	29/75	0.195.
BMI	23.6 (2.16)	24.3 (3.21)	0.373
Pre VAS	7.29 (0.99)	5.48 (1.20)	< 0.001^*^
Pre MEPS	59.1 (5.37)	61.8 (5.93)	0.061
Pre Quick DASH	56.4 (6.49)	54.6 (7.06)	0.333

*VAS* Visual analogue scale, *MEPS* Mayo Elbow Performance index Score, *Quick DASH* Quick Disabilities of the Arm, Shoulder and Hand Score

^*^P < 0.05

## Discussion

An elbow with significant instability can be detected via conventional physical examinations. However, it is difficult to demonstrate chronic ligament injury of subtle instability alone in the awake patient because of muscular restraints. Thus, EUA with muscle relaxation should be performed in these patients [16, 17]. On EUA, pathologic gapping of the lateral joint in the AP stress view or posterior translation of the radial head in the lateral stress view may provide evidence of lateral elbow instabilities and was applied in the current study [16]. Previously, EUA was not usually performed in lateral epicondylitis, and only arthroscopic EUA was reported [6]. In this study of 40 patients, 13 patients with dehiscence between 3 and 6 mm showed slight instability, and 2 patients with dehiscence over 6 mm showed severe instability. Arthroscopic examination had some potential advantages because there was no need for radiation and it was easy to perform anytime during the surgery. However, this procedure had a disadvantage in that surgeons could identify dehiscence on varus or valgus stress tests alone and obtain little information about posterolateral instability. Moreover, joint distension with saline can influence the tension of the capsule and ligament, causing some difficulties in analyzing the stability. Fluoroscopic EUA poses a risk of radiation exposure, and it might be difficult to note minor instability. However, it is possible to distinguish posterolateral instability from simple coronal instability, and it is easy to perform without any special technique. Moreover, it can provide more intuitive images of instability. Considering these advantages and disadvantages, the surgeon must decide which method to choose in identifying instability in EUA.

While few reports have been published about combined instability in EUA, there are several reports about associated ligament injuries with lateral epicondylitis identified via MRI. Potter et al. reported that 4 of 20 patients had associated radial collateral ligament injuries with MRI and those lesions were confirmed during surgery [17]. More recent studies have reported a positive correlation between the severity of common extensor tendon injury and that of LUCL injury [1, 2]. Moreover, in a study comparing 9 patients and 9 asymptomatic volunteers, MRI was reported to be effective in determining ligament abnormality in patients with subtle elbow instability [18]. Thus, when suspected, it would be helpful to perform an MRI of the elbow. However, in elbows with lateral epicondylitis, MRI was not completely correlated with EUA and operative findings. In our study, 15 of the 28 abnormal elbow MRIs showed subtle instability (positive predictive value, 53.6%), while 81 of the 82 normal MRIs did not show subtle instability (negative predictive value, 98.7%). Considering the relatively low positive and high negative predictive value, we think MRI alone is not recommended in determining the surgical procedure, and EUA should be performed when there are ligament abnormalities in MRI.

It is clear that an unstable elbow with significant instability should be stabilized because detrimental articular contact in the elbow joint usually leads to irreversible cartilage injury [19]. However, whether subtle instability during EUA should be surgically treated is controversial. Aoki et al. reported that a patient with mild lateral instability had a good outcome even with conventional debridement [20]. Coleman et al. reported that synovial fistulae developed in 2 patients postoperatively [21]. One patient with an irreparable defect in the elbow capsule during surgery had to undergo an anconeus flap operation, and the other was managed conservatively. There was no mention about instability in these 2 patients. However, Kalainov et al. reported that 3 patients with large fistulae with instability had to undergo LUCL reconstruction [5]. Moreover, several authors have recommended surgical correction for instability, which was only identified in EUA [16, 18]. We believe that such instabilities, identified during EUA, should preferably be corrected; however, evidence supporting this proposition is still lacking. It might also be unclear which procedure should be selected for subtle instability. Ligament repair, including imbrication or tensioning, could be an option [22]; however, ligamentous tissue often showed poor quality in cases of chronic posterolateral instability, and ligament reconstruction might provide more consistent results [18, 23, 24]. In our study, 13 patients underwent ligament reconstruction because ligament repair was not feasible. Thus, if surgeons decide to address subtle instability, they should prepare for ligament reconstruction as well as repair.

We investigated preoperative factors that were known to affect the prognosis of lateral epicondylitis, to determine which of the preoperative clinical data was different between the two groups. In conservative treatments, demographic factors including age and gender were reported to have no significant relationship with the prognosis of lateral epicondylitis, while the effect of site involvement was unclear. Manual work, high strain at work, long duration of symptoms, multiple musculoskeletal complaints, and low socioeconomic status were reported as poor prognostic factors at 12 months of treatment [7, 12, 14]. After surgical treatments, high baseline pain, sudden onset of symptoms, long duration, morbid obesity, smoking, a history of multiple corticosteroid injections (≥3), and young age were reported as potential risk factors for poor surgical outcome [13, 15]. The effect of gender on outcome was considered controversial [13, 25]. Some authors even mentioned previous corticosteroid injection as a cause of ligament tear [5, 26], and a history of multiple corticosteroid injections (≥3) was reported as the most significant risk factor for surgical treatment failure [17]. Among those factors, our results suggest that a high level of pain and history of multiple corticosteroid injections (≥3) are preoperative characteristics of lateral epicondylitis with subtle instability. However, patient’s recollection of how many injections they received was inaccurate in our study, so we could not perform analysis based on the exact number of injections. Moreover, since the VAS score did not indicate the absolute value of pain, it was difficult to provide cut-off values for pain level predictive of subtle instability.

The present study revealed that some patients (13.9% of the patients that underwent surgery for chronic lateral epicondylitis) showed varus or posterolateral subtle instability during fluoroscopic EUA. To our knowledge, our study is the first to report the predictive value of MRI for subtle instability. Nevertheless, our study had several limitations. First, the number of patients in the unstable group was relatively small because subtle instability in EUA is uncommon in lateral epicondylitis. Therefore, results such as risk ratio could not be presented. Second, the force applied during the stress test was not identical. Thus, it was impossible to analyze the joint widening quantitatively. Third, exploration of the LCL complex was not performed in the stable group. Thus, the relationship between the structural status of ligaments and MRI findings was not revealed in this study. Further study should include a larger number of patients and the same force for stress test to overcome these limitations.

## Conclusion

Subtle instability resulting from chronic LCL complex injury was noted in elbows with lateral epicondylitis. This is difficult to detect in conventional physical examination but can be easily visualized in fluoroscopic EUA. Preoperative MRIs can be used to exclude subtle instability with its high negative predictive value. The unstable group tended to have higher VAS scores than the stable group, and a history of multiple corticosteroid injections (≥3) was an indication of subtle instability. Although it was unclear whether surgical treatment of the LCL complex should be performed, surgeons should consider checking for subtle instability, especially when patients have a history of multiple corticosteroid injections (≥3) or severe pain and MRI indicates instability.

## Abbreviations

BMI: Body mass index
ECRB: Extensor carpi radialis brevis
EUA: Examination under anesthesia
LCL: Lateral collateral ligament
LUCL: Lateral ulnar collateral ligament
MEPS: Mayo Elbow Performance Score
MRI: Magnetic resonance imaging
Quick DASH: Quick Disabilities of the Arm, Shoulder, and Hand
SD: Standard deviation
VAS: Visual analogue scale
